# Full-Thickness Eyelid Lesions in Sarcoidosis

**DOI:** 10.1155/2013/579121

**Published:** 2013-05-09

**Authors:** Megan E. Collins, Vesna Petronic-Rosic, Nadera J. Sweiss, Marcus M. Marcet

**Affiliations:** ^1^Department of Ophthalmology, University of Wisconsin, Madison, WI, USA; ^2^Section of Dermatology, University of Chicago, Chicago, IL, USA; ^3^Section of Rheumatology, University of Illinois, Chicago, IL, USA; ^4^Department of Ophthalmology, University of Hong Kong, Cyberport, Hong Kong

## Abstract

Eyelid involvement in sarcoidosis is very rare. A search of the medical literature indicates one previous report of sarcoidosis with destructive eyelid lesions. We describe the case of a 50-year-old woman with severe systemic sarcoidosis, which included her eyelids. To our knowledge, the case presented herein represents the first to show the full-thickness histopathology of destructive eyelid lesions in sarcoidosis.

## 1. Introduction

Sarcoidosis is a multisystem inflammatory disease of unknown etiology characterized histopathologically by noncaseating granulomas. The condition has a female predilection with a peak incidence in the 3rd and 5th decades of life. Pulmonary involvement is the most common manifestation. Common extrapulmonary manifestations include the skin, central nervous system, liver, kidney, and musculoskeletal system. Furthermore, sarcoidosis can affect the heart, peripheral nervous system, salivary glands, eye, and ocular adnexa. Ocular involvement occurs in 25%–60% of patients with systemic sarcoidosis [1, 2]. While the disease can involve any ocular tissue, anterior uveitis and lacrimal gland involvement are the most frequently reported findings [3]. Eyelid manifestations of sarcoidosis include “millet seed” nodules, ulcerated nodules, plaques, and swollen eyelids [4]. Eyelid involvement in sarcoidosis is very rare. To our knowledge, there is only one previous report in the literature that describes full-thickness eyelid involvement. Our case is the first to show the full-thickness histopathology of destructive eyelid lesions in sarcoidosis.

## 2. Case Report

A 50-year-old female with severe systemic sarcoidosis, including pulmonary, skin, and joint disease, presented for evaluation of ocular involvement. The patient had noted eyelid nodules for over 5 years; however, she recently developed eyelid deformities, tearing, and eye irritation.

**Figure 1:**
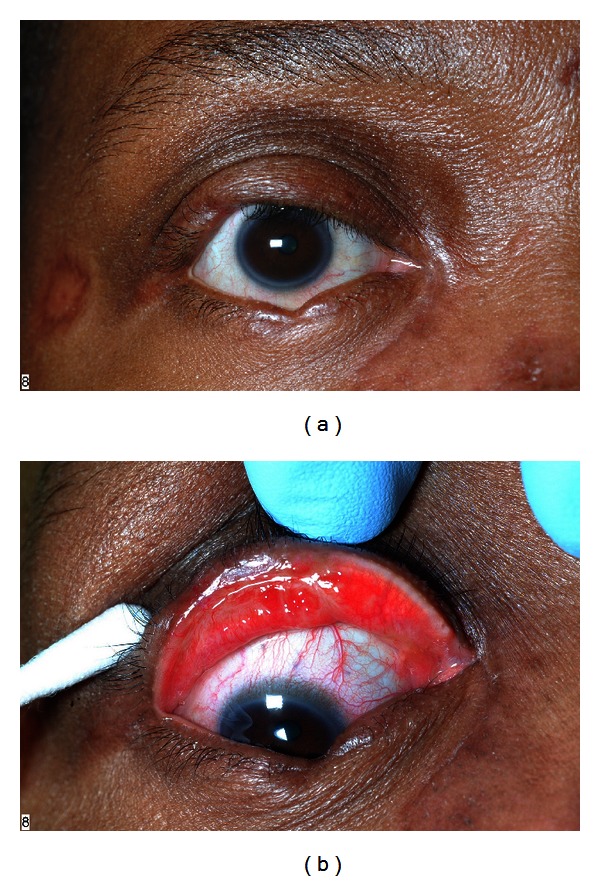
(a) Preoperative photograph of the patient showing eyelid notching, entropion, and trichiasis. Multiple sarcoid nodules on the face are present. (b) Preoperative photograph of everted right upper eyelid showing focal trichiasis, cicatricial entropion, and thickened hyperemic conjunctival scarring. Inspissated meibomian glands are seen in the medial third of the eyelid. There is grossly apparent loss of the meibomian glands in the lateral two thirds of the eyelid.

On examination, she had notching of her upper and lower eyelids and extensive scarring of the posterior lamella leading to entropion and focal trichiasis (Figure 1). Slit lamp examination showed complete inspissation of the meibomian glands and superficial keratitis corresponding with misdirected eyelashes. The remainder of the anterior and posterior segment examination was within normal limits. Medical therapy did not adequately control the patient's symptoms.

**Figure 2:**
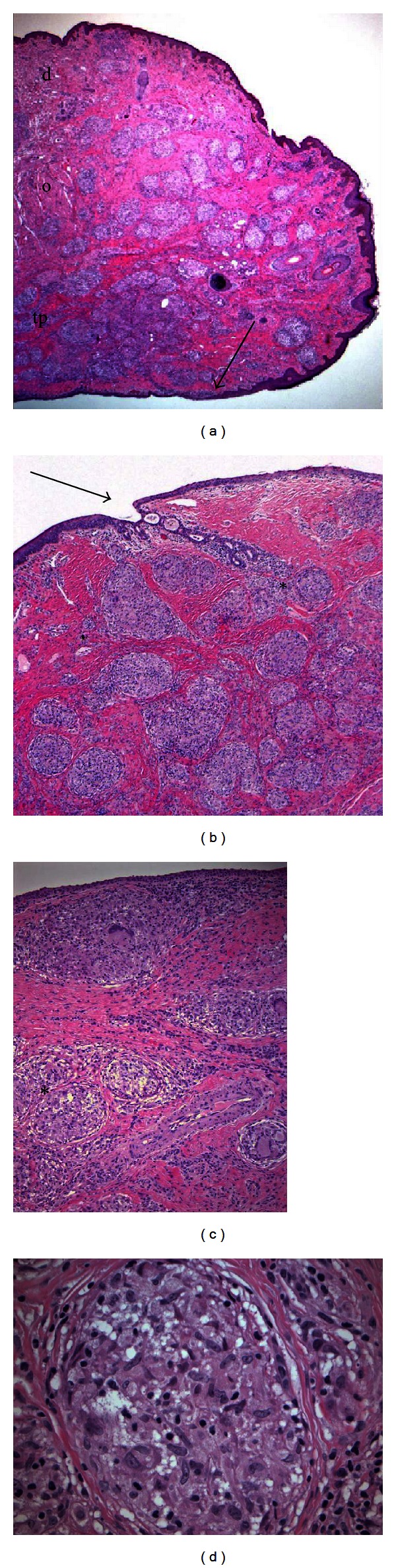
(a) Photomicrograph of sagittally sectioned full-thickness wedge resection showing a thickened eyelid with noncaseating granulomas infiltrating dermis, orbicularis oculi muscle, and tarsus. The mucocutaneous junction is seen (arrow). There is absence of the meibomian glands (hematoxylin-eosin, ×20). The eyelid is designed as follows: tp = tarsal plate; o = orbicularis oculi muscle; d = dermis. (b) Cross-section through the meibomian gland orifice (arrow) with sarcoidal obliteration of the meibomian glands (asterisk) (hematoxylin-eosin, ×100). (c) Tarsal (asterisk) granulomas with multinucleated giant cells and normal overlying palpebral conjunctiva (hematoxylin-eosin, ×200). (d) Higher magnification view of tarsus showing noncaseating granuloma containing epitheliod histiocytes (hematoxylin-eosin ×400).

The patient subsequently underwent full-thickness wedge resections in both upper eyelids and her right lower eyelid resulting in symptomatic improvement and elimination of the localized areas of trichiasis. Histopathologic evaluation of all three full-thickness wedge resections showed typical “naked,” noncaseating granulomas composed of epitheliod histiocytes and multinucleated giant cells (Figure 2). Serial sections showed extensive destruction of both the anterior and posterior lamellar anatomies, including derangement of the tarsal architecture, obliteration of the meibomian glands, and infiltration of the dermis and orbicularis oculi muscle. Entropic migration of the mucocutaneous junction and eyelash misdirection were also seen. Ziehl-Neelsen and Gomori's methenamine silver stains were negative for microorganisms.

## 3. Discussion

 Early reports have limited discussion of the incidence of eyelid involvement in sarcoidosis [3, 5]. Eyelid manifestations of sarcoidosis include “millet seed” nodules, ulcerated nodules, plaques, and swollen eyelids [4]. In 1986, Jabs and Johns described eyelid nodules in 5/183 patients (0.27%) with systemic sarcoidosis [1]; however, a 2007 review of 26 patients with sarcoidosis and orbital disease found eyelid involvement in 11.5% of patients [2]. The actual incidence of eyelid involvement remains uncertain. In addition, limited data has been published on the correlation of eyelid involvement and disease severity. For example, our patient had severe systemic sarcoidosis resistant to aggressive immunosuppressive and immunomodulating therapy.

A Pubmed literature search (http://www.ncbi.nlm.nih.gov/pubmed/, accessed 10 March 2013) of “eyelid and sarcoid” indicates one previous case [6] of involvement of both the anterior and posterior lamellae of the eyelids. In 2001 Moin et al. first described destructive full-thickness sarcoidosis lesions of the eyelids [6]. The authors showed a high magnification image of a small segment of tissue; however, an oriented figure with the full-thickness microscopic anatomy of the eyelid was absent [6]. To our knowledge, a full-thickness histologic depiction of an eyelid showing destructive lesions in sarcoidosis has not been previously published. In addition, the complete obliteration of the eyelid meibomian glands by the sarcoidosis granulomatous infiltrates seen in our case represents new findings. Dry eyes in patients with sarcoidosis are often attributed to sarcoid infiltration of the lacrimal gland. Based on our findings, sarcoid destruction of the meibomian glands may represent an additional etiology for dry eyes in sarcoidosis patients with eyelid involvement.
